# 

**Published:** 1845-06-01

**Authors:** 


### INDEX TO VOLUME FIRST.

Aortitis, case of with Autopsy, by G. N. Burwell, M. D. - - P. 9
Anaplasty, by Prof. Hamilton, - 62, 135
Accoucheur, letters by 110, 131, 160, 176, 247
Aneurism by compression, treatment of 163
Atmosphere, heated therapeutic action of - - - - - - 192
Ammonia phosphat in gout, and rheumatism, - - - - - - 234
Analyses of Mineral Waters near Buffalo, by Lieut. Long, M. D. 197
Antidote to Arsenic, - - - 283
Anatomical Remembrancer, - - 280
Burwell, G. N., M. D., on Aortitis, 9
On singular attempt at Suicide, - 36
On Typhoid Fever, - - - 156
On Epidemic at North Boston, - 91
Beech, J. H. M. D., on Needle found in heart of a Cow, - - - 177
Bigelow, H. J. M. D., Address, - 284
Baltimore College of Dental Surgery, 284
Braithwaite's Retrospect, - - - 281
Backus, Dr. reports in Senate of New-York, - - - - - - 279, 280
Conium, dose of extract, - - 16
Cyclopædia of Practical Medicine, 18
Copland's Med. Dictionary, 18, 139, 281
Cleveland Marine Hospital, - - 18
Cranium Fracture of, case by Darwin Colver, M. D. - - - - - 35
Cystotomia, case by J. P. White, M. D. - - - - - - 104
Conium, Physiological effects of 120, 232
Convention, National Medical - 120
Convention, appointment of Delegates by State Society, - - 237
Convention, action of State Medical Society, - - - - - - 253
Channing, Prof. Walter Lecture by 189
Catalogues, Fictitious of Medical Students, - - - - - - 238
Cyanosis of new born Infants, - - 214
Citrate of Iron in Chlorosis, - - 277
Dislocation of large joints, reduction of by power from twisted rope, - 16
Diabetes Mellitus, - - - - 56
Drake, Daniel Prof. contemplated Work by - - - - - - 214
European tour, by Prof. Hamilton, pages 3, 25, 49, 73, 97, 121, 145, 170, 200, 225, 241
Erie Co. Med. Society, semi-annual meeting of - - - - - 47
Erie Co. Med. Society annual meeting of - - - - - - - 213
Electro-Physiology, - - - - 164
Exchange Journals, - - 166, 272
Evacuation, green in Children, - 191
Eau Brocchieri, - - - - 240
Erysipelas, Epidemic in Williamsville, by Wm. Van Pelt, M. D. - - 193
Erysipelas, Epidemic in Springville, by John G. House, M. D. - - 154
Ellis' Medical Formulary—correction, 284
Fever, Epidemic at North Boston, by Austin Flint, M. D. - - - 91
Fever, Opium large doses in - - 113
Fever, Pathology of by Editor, - - 133
Fever, Remittent, Typhus and Typhoid distinctive characters of, by Editor, - - - - - - 178
Fever, Typhoid case of by G. N. Burwell, M. D. - - - - - 156
Fees, Medical - - 115, 141, 168
Forensic Medicine, by Guy and Lee, 236
French Medical Congress, - - 256
Gastrotomy, case of - - - 70
Hamilton, Prof. Notes of an European Tour, 2, 25, 49, 73, 97, 121, 145, 170, 200, 225, 241
Hamilton, lecture on Phrenology, - 19
Hamilton, on Strabismus, - - 167
Hamilton, on Anaplasty, - - 62, 135
Hamilton, Report of Surgical cases, 244
Hydrocianic Acid, antidote for - - 11
Homœopathy, - - - - - 142
Hernia omental, case by A. S. Sprague, M. D. - - - - - - - 153
Homicide, Judicial - - - 239
Hastings, B. F. M. D., Surgical cases, . . . . . . 248
House, John G. M. D., on Epidemic Erysipelas, - - - - - 154
Intestine, removal of 17 inches of, with recovery of patient, - - 16
Irritation from extraneous substances, by Wm. Treat, M. D. - - 60
Iodide of Iron, - - - - 141
Journals in New-York, - - - 143
Loomis, H. N. M. D., cases of twins, 15
Long, Lieut. E. R. M. D., Analysis of Pie-Plant, - - - - 37
Long, case of Pleuro-Pneumonitis, 32
Long, reports of sick at Buffalo Barracks, - - - - - - - 126
Long, Analyses of Mineral Waters near Buffalo, - - - - 197
Long, Obituary of - - - - 263
Liebig in Great Britain, - - - 69
Lewis, Winslow, M.D. Surgical op'n by 71
Luxations, - - - - - 85
Lunatic Asylums, reports of - - 279
Marine Hospital at Cleveland, - - 18
Malpractice, legal responsibility for 39
Morbis Cordis, case of by Editor, 111
Medico-Chirurgical Review, - - 144
Mercury, ferruginous pill of - - 144

Medical Remembrancer, - - 167
Medical Legislation, - - 137, 168
Medical reform in England, - - 69
Medical reform, - - 212, 229, 249
Medical University, - - - 215
Medical Schools, - - - 70, 117
Medicines, Spurious - - - 232
New-York Journal of Medicine, - 19
New-York State Medical Society, transactions of 1845, - - - 43
Needle in the heart of a Cow, - - 177
Nipple, artificial, Pratt's - - - 192
National Convention, - - - 120
National appointment of Delegates by State Society, - . - 237
National, action of State Society upon - - - - - - - 253
Needle, extraction of from hand, - 215
Oxalic Acid in Pie-plant, - 19
Oedema of Glottis, case of, by J. P. White, M. D. - - - - 30
Ova, periodic discharge of - - 60
Okee-Chobee, battle wounded at, report of, by R. C. Wood, M. D. U. S. Army, - - - - - 80
Pleuro-pneumonitis, case of by Lieut. Long, M. D. and Editor, - - 32
Pie-plant, analysis of by Lieut. Long, M. D. - - - - - - - - 37
Pathology, treatise by Grisolle, - - 95
Pthisis in New-Orleans, - - - 96
Placenta, delivery of before the child in placenta-prævia, - - - 114
Physicians in Buffalo, - - - 162
Placenta-prævia, case of treated on Prof. Simpson's method, - - 166
Petrified human body, - - - 192
Phrenology, Lectureship on - - 283
Physicians in London, - - - 283
Paine's, Prof. defence of Medical Profession, - - - - - - 278
Quinia in Typhoid and all other Fevers, - - - - - 66
Quinia in Chlorosis and other anœmic states, - - - - - - 277
Quackery, Medical by Wm. Treat, M. D. - - - - - - 173
Rheumatism, acute cases of treated with Nitras Potassæ, by Alden S. Sprague, M, D. - - - - 8
Reform, Medical - - 212, 229, 249
Rush Medical College, - - - 70
Richardson, Dr. Wm. H. Obituary, 119
Reports of sick at Buffalo Barracks, 126
Ricord on Veneral diseases, - 204, 218
Ranking's Abstract, - - - 280
Sprague, Alden S. M. D., cases acute Rheumatism, - - - - - 8
Sprague, case Lithotomy, - - 34
Sprague, case Fracture of Skull, - 108
Sprague, case Omental Hernia, - - 153
Suicide, singular attempt at by a Lunatic, - - - - - - - 36
Surgery, operative elements of by Velpeau and Mott, - - - 46
Surgery, principles of by Miller, - 46
Surgery, first lines by Cooper and Parker, - - - - - - 47
Sanitary condition of New-York, by Griscom, - - - - - - 67
Spencer, Thomas M. D., on Vital Chemistry, - - - - - 67
Surgeon, General's Cicular, - - 71
Strabismus, Monograph by Prof. Hamilton, - - - - - - - 167
Surgical cases, by Prof. Hamilton, 244
Surgical, by B. F. Hastings, M. D. - - - - - - - - - 248
Seminal Emissions, Involuntary, treatment of - - - - - 275
Syphilis Tertiary, Prof. Parker's Clinique, - - - - - - - 276
Treat, Wm. M. D., on Local Irritation, - - - - - - 60
Treat, on Medical Quackery, - - 173
Twins, cases of at different stages of development, H. N. Loomis, M. D. 15
Transcendental Anatomy, - - 17
Trowbridge, Josiah M. D., Address before Buffalo Med. Association, 88
Trowbridge on Turpeth Mineral in Croup, - - - - - 270
Typhoid Fever, case by G. N. Burwell, M. D. - - - - - 156
Trephining Operation, by Prof. Brainard, - - - - - - 168
Transactions, College of Physicians, Philadelphia, - - - - 187
Transactions of State Medical Society 1845, - - - - - - 43
Tonsils, Hemorrhage after excision, mode of arresting, by Prof. Hamilton, - - - - - - - 277
Test for Bile and Sugar, - - - 282
Uterus, Extraction of in an effort to remove Placenta, - - - 39
Venereal Diseases, by Ricord, 204, 218
Velpeau's Surgery, - - - 277
Van Pelt, Wm. M. D., on Epidemic Erysipelas, - - - - - 193
Vaccination after exposure to Small-Pox, - - - - - - - 281
White, James P., M. D., case of Œdema of Glottis, - - - 130
White, case of Cystotomia, - - 04
White, Rupture of Ovarian Sac, - 151
Willoughby Medical College, appointment in - - - - - - 71
Wood, R. C. M. D., U. S. Army, report of wounded at the battle of Okee-Chobee, . - - - 80
Wakefulness, protracted - - 96
Water Cure by Sir E. L. Bulwer, 183
Waterman, Wm. B. and State of New-York, - - - - 190
Works of Hippocrates and Galen, 214

☞ We would direct the attention of the Profession to the advertisement of John C. Sieffert which will be found on the advertising sheet. Mr. S. served his apprenticeship in Strasburg and Berlin, and has been established in Buffalo as an instrument maker, for the last ten years.

His instruments, for excellence and beauty of finish, will compare with those manufactured in our larger cities or imported from Europe. He has given great satisfaction in this city and vicinity, and we cordially commend him to the farther patronage of the profession.

---

### To Readers and Correspondents.

Communications have been received from Drs. White, Long, and Colvin, which are on file for publication. From the alacrity which our brethren in this city have manifested in furnishing contributions, and the expressions of interest which have been received since our circular was issued, we are under no immediate apprehensions of any lack of original matter, but rather that our limits are disproportionately small for the amount which may be obtained. We hope that members of the profession in the Western part of this State, and in the Western States bordering on the Lakes, with whom communication is so easy and expeditious, will co-operate with us. We shall be glad to receive communications from them, only asking in addition, that they will endeavor to extend our circulation, so that if circumstances require it, we may be able to enlarge our limits sufficiently to do justice to our readers and correspondents.

The transactions of the New-York State Medical Society at their annual meeting in February last, have been received, but too late for any reviewal in this number.

We publish in this number the Meteorological Register kept at the U. S. Barracks in this city. For this we are indebted to the kindness of Dr. Wood, Surgeon at the post, who in our behalf applied for and obtained permission from the Surgeon General to supply a transcript of his monthly official Meteorological report for this Journal. We expect to continue its publication regularly, so that our patrons will have a Meteorological register for the entire year. This will often be useful for reference ; and as connected with the etiology of disease may prove to be highly valuable.

---

☞ We have issued a large edition of the present number of the Journal and it will be forwarded to many individuals as a specimen of the work, with the hope that if it meet their approval they will forward their names with the amount of subscription. To those who do not thus signify their intention to patronize it, it will not, of course, be continued. The necessity of remitting the subscription price *in advance*, when the amount is so small must be apparent ; and to avoid all misconstruction we take occasion to state distinctly that the terms in this respect will in no instance be deviated from.

Remittances can be made without expense to subscribers or publisher, through Postmasters.

